# Failure to complete adjuvant chemotherapy is associated with adverse survival in stage III colon cancer patients

**DOI:** 10.1038/sj.bjc.6603627

**Published:** 2007-02-13

**Authors:** M Morris, C Platell, L Fritschi, B Iacopetta

**Affiliations:** 1 School of Surgery and Pathology, University of Western Australia, Nedlands 6009, Australia; 2 The St John of God CRC Unit, Perth, Western Australia, Nedlands 6009, Australia; 3 Western Australian Institute for Medical Research, University of Western Australia, Nedlands 6009, Australia

**Keywords:** colon cancer, health services, mayo regime, toxicity, 5-fluorouracil

## Abstract

Two recent North American studies have shown that completion of 5-fluorouracil (5FU)-based adjuvant chemotherapy is a major prognostic factor for the survival of elderly stage III colon cancer patients. The aim of the present study was to confirm this finding in a population-based series from Australia. The study cohort comprised 851 stage III colon cancer patients treated by surgery alone and 461 who initiated the Mayo chemotherapy regime. One-third of patients who initiated chemotherapy failed to complete more than three cycles of treatment. Independent predictors for failure to complete were treatment in district or rural hospitals, low socioeconomic index and treatment by a low-volume surgeon. Patients who failed to complete chemotherapy showed worse cancer-specific survival compared not only to those who completed treatment (HR=2.24; 95% confidence interval (CI) (1.66–3.03), *P*<0.001) but also to those treated by surgery alone (HR=1.37; 95% CI (1.09–1.72), *P*=0.008). The current and previous studies demonstrate the importance of completing adjuvant 5-FU-based chemotherapy for colon cancer. Further prospective studies are required to identify better the physiological and socioeconomic factors responsible for failure to complete chemotherapy so that appropriate improvements in health service delivery can be made.

Randomised controlled trials conducted in the 1980s demonstrated that 5-fluorouracil (5FU)-based chemotherapy resulted in a 10% absolute improvement in 5-year survival for stage III CRC patients (Moertel *et al*, 1990). As a result of these trials the National Institutes of Health recommended in 1990 the routine administration of FU-based adjuvant chemotherapy for medically fit patients with completely resected stage III CRC (NIH Consensus Conference, 1990). In the early 1990s, adjuvant chemotherapy with 5FU was used in combination with levamisole or leucovorin and regimes varied from 6 to 12 months in length. By 1995, the standard of care in many countries, including Australia, had become the Mayo regime of intravenous 5FU/leucovorin for 6 months.

Randomised controlled clinical trials generally analyse the benefits of treatment in patient cohorts with few comorbidities. Participants in the earlier randomised clinical trials for CRC were highly selected and most patients were aged <70 years. These do not accurately represent all patients who may ultimately become candidates for treatment in the general population. Nevertheless, several reports have recently documented a similar degree of survival benefit from 5FU in older patient groups from a population-based setting (Iwashyna and Lamont, 2002; Sundararajan *et al*, 2002; Jessup *et al*, 2005; Dobie *et al*, 2006; Neugut *et al*, 2006). These results support earlier evidence from randomised control trials and clearly establish benefit from 5FU-based adjuvant chemotherapy in stage III colon cancer.

Two recently published studies using the SEER database examined the early termination of adjuvant chemotherapy regimes in the elderly population in relation to survival (Dobie *et al*, 2006; Neugut *et al*, 2006). These papers reported that patients who failed to complete 5FU-based chemotherapy showed significantly worse survival compared to those who completed the treatment. Confirmation of the findings with respect to completion of treatment has important implications for the delivery of effective healthcare to patients with colon cancer. This paper examines the effect on survival of failure to complete adjuvant chemotherapy in a population-based cohort that includes patients of all ages and who were treated exclusively with the Mayo regime.

## METHODS

### Study population

Pathology records from the four major hospitals in Western Australia were used to identify patients diagnosed with CRC during the period 1994–2001 inclusive. The pathology services at these hospitals also process specimens from minor district and country hospitals. This patient list was crosschecked with the Cancer Registry of Western Australia. Approximately 90% of all CRC patients who underwent surgical resection were identified for the population of Western Australia, comprising 1.8–2 million people over the study period. Tumour stage was classified according to the current American Joint Committee on Cancer guidelines (*AJCC Cancer Staging Manual*, 6th edn, 2002). A total of 2024 patients with CRC fulfilled the criteria for stage III CRC and 1404 of these had colonic carcinoma. All cases showed clear margins (R0 resections). Rectal carcinomas were defined as originating within 12 cm of the anal verge and these were excluded from the analysis. Information on pathological variables was obtained from the histopathology reports. Perforation was considered to be present if noted by the surgeon at operation or on histopathological review of the specimen. Clinical records were used to classify tumours presenting with obstruction. Anatomical site of the tumour was crosschecked with information from admission and procedure records. Colonic cancers were subclassified as being proximal or distal to, and including, the splenic flexure.

Surgical caseload was defined as low (⩽10), medium (11–50) and high (>50) for stage II and III colon cancer resections over the 8-year study period. Hospitals were classified as teaching (university affiliated), private (fee for service), district (non-teaching and non-private institutions located in the metropolitan region of Perth) or rural (non-metropolitan). Each patient's post code address was obtained from the West Australian Cancer Registry database and this was linked to Socio-Economic Indexes for Areas (advantaged/disadvantaged and economic resources) obtained from the 2001 Australian census (Australian Bureau of Statistics, 2001). Patients with an advantaged/disadvantaged score of 1 were the most deprived quintile in socioeconomic terms, whereas a score of 5 corresponds to the most advantaged group. Patients with a score of 1 for economic resources had the least financial resources, whereas those with a score of 5 had the most. Ethics approval for the project was obtained from individual hospital Human Research Ethics Committees, the University of Western Australia and the Confidentiality of Health Information Committee.

### Adjuvant chemotherapy

Procedure codes from the morbidity database of the Data Linkage Unit, Health Department of Western Australia, were used to identify patients who began chemotherapy within 120 days of resection. The adjuvant chemotherapy regimes used in Western Australia varied during the study period. Cases were individually reviewed using hospital records and only those patients (*n*=461) who received the Mayo regime were included in the study. A total of 92 patients who received the Roswell or other regimes were excluded. Patients who received chemotherapy for a recurrence were also documented (*n*=150). Less than 15 doses administered were defined as ⩽3 cycles (*n*=156) and 16–30 doses as 4–6 cycles (*n*=305). Therefore, the study cohort (*n*=1312) comprised 851 patients treated by surgery alone and 461 who initiated the Mayo chemotherapy regime.

### Survival data

Mortality data were obtained from the Death Registry of the Health Department of Western Australia. Death reports were reviewed individually and classified as death due to colon cancer or from other causes. The perioperative mortality rate (4.8%) was defined as death within 30 days of surgery. At the end of the study period, 155 (11.8%) patients died from unrelated causes and 657 (50.1%) from recurrence of colonic cancer. Of the 461 patients who initiated adjuvant chemotherapy, three patients died as a result of chemotherapy treatment (0.65%). Sepsis and pancytopaenia were responsible for two deaths and one patient died of gastrointestinal haemorrhage secondary to a duodenal ulceration. One other patient died from a cerebrovascular accident while on chemotherapy 3 months after resection. Survival time was calculated from the date of diagnosis to date of death from cancer or 1 March 2006, whichever came first. This enabled cancer-specific and overall survival to be evaluated. The mean length of follow-up was 52 months and the median was 36 months (range 0–147 months).

### Statistical analysis

Chi square analysis was used to identify factors influencing the rates of chemotherapy initiation and completion. A multiple logistic regression model in which each demographic, pathological and clinical variable listed in Table 1 was adjusted for all others was used to estimate odds ratios and 95% confidence intervals (CIs) for independent predictors of chemotherapy initiation or completion. Survival analysis was conducted using both unadjusted Kaplan–Meier analysis and Cox proportional hazards regression. The log-rank test was used to determine the significance for Kaplan–Meier analysis. A Cox proportional hazards regression model was developed for survival in which each variable was adjusted for all others. Statistical significance was deemed if *P*<0.05.

## RESULTS

**Figure 1:**
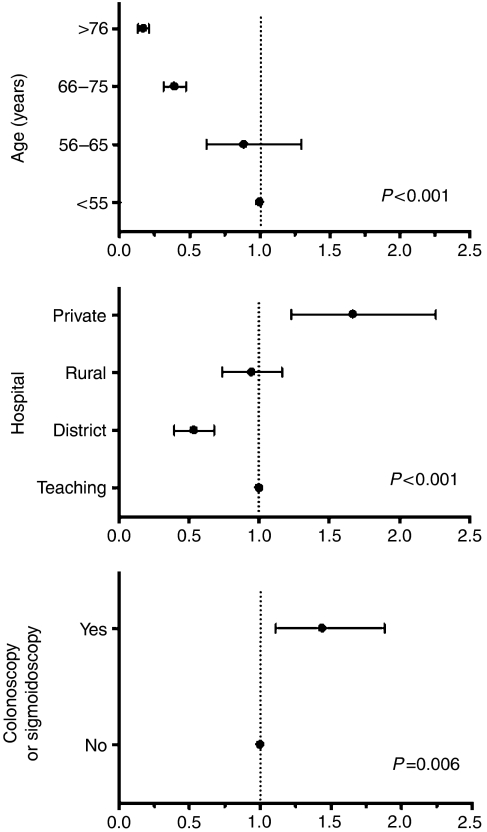
Predictors for the initiation of 5FU adjuvant chemotherapy in stage III colon cancer patients adjusted in multivariate analysis.

In this population-based cohort, just over one-third of stage III colon cancer patients initiated chemotherapy using the Mayo regime (Tables 1 and 2). From 1997 to 2001, the rate remained steady at approximately 40% of cases. This is probably reflective of stabilisation of surgical referral and oncological practice following the initial period of 5FU chemotherapy implementation. As expected, chemotherapy use declined with increasing age. Patients treated in private hospitals and those whose tumours were detected by colonoscopy or sigmoidoscopy were more likely to initiate chemotherapy. These same three factors were found in multivariate analysis to be independent predictors for the initiation of chemotherapy (Figure 1). None of the pathological variables were associated with the commencement of chemotherapy.

**Figure 2:**
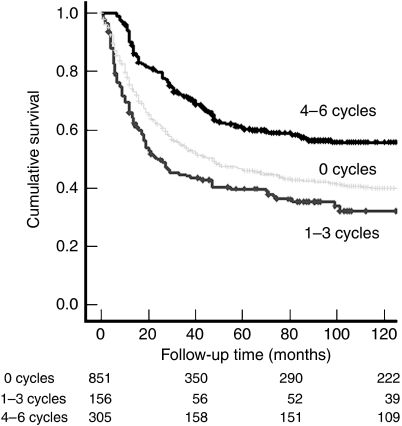
Kaplan–Meier survival analysis for stage III colon cancer patients treated with surgery alone (0 cycles, light grey), 1–3 cycles (incomplete chemotherapy, dark grey) or 4–6 cycles (complete chemotherapy, black) of 5FU adjuvant chemotherapy using the Mayo regime. Log-rank test: *P*=0.021 for incomplete chemotherapy *vs* surgery alone *P*<0.0001 for complete chemotherapy *vs* surgery alone; *P*<0.0001 for complete chemotherapy *vs* incomplete chemotherapy.

The survival of patients who initiated chemotherapy is shown in Table 3 according to the number of cycles received. Compared to patients treated by surgery alone, those who received only one cycle of chemotherapy showed significantly worse survival. A trend for worse survival was also observed for patients treated with two or three cycles. In contrast, patients treated with four, five or six cycles showed better survival than those treated by surgery alone. On the basis of these results and for this study, patients who received 4–6 cycles were classified as having completed chemotherapy, whereas those treated with 1–3 cycles were deemed not to have completed this treatment. The former group was estimated to have a 30% survival advantage and the latter group a 40% survival disadvantage compared to patients treated by surgery alone (Table 3 and Figure 2).

Two-thirds of patients who initiated chemotherapy completed 4–6 cycles of treatment (Tables 4 and 5). Factors associated with higher rates of completion were N1 nodal status, high surgeon caseload, treatment in teaching and private hospitals and high socioeconomic indices. Multivariate analysis revealed that independent predictors for completion of chemotherapy were the type of treatment hospital, high socioeconomic index and high surgical volume (Table 6). Females showed a trend for less likelihood of completion (*P*=0.08).

## DISCUSSION

Two recent studies using SEER data reported that stage III colon cancer patients who failed to complete 5FU chemotherapy showed worse survival compared to those who completed the regimen (Dobie *et al*, 2006; Neugut *et al*, 2006). The present investigation of an Australian population-based cohort of stage III colon cancer confirms the findings of the North American groups. Moreover, the current study also found that patients who initiated but did not complete chemotherapy (1–3 cycles received) showed significantly worse survival than patients treated by surgery alone (Table 3).

There were several differences in study design between the current investigation and the North American reports. Patients of all ages were included here, whereas only >65-year-old patients were investigated previously. Despite the younger cohort, only 35% of patients initiated chemotherapy (Tables 1 and 2) compared to 54 and 55% for the SEER-derived cohorts (Dobie *et al*, 2006; Neugut *et al*, 2006). The completion rate for patients who initiated chemotherapy in the present investigation (66%,Tables 4 and 5) was comparable to the North American report (64%) from the same study period (Neugut *et al*, 2006). Both were slightly lower than the second North American report (78%), which investigated an earlier study period, included both 12 and 6 month regimes and presented 3-year cancer-specific mortality data (Dobie *et al*, 2006). Although the present study had a smaller sample size, individual patient records were reviewed for pathology, chemotherapy regime and cause of death.

In spite of these minor differences in study design, all three investigations have observed a significant survival advantage associated with the completion of chemotherapy. The survival advantage was estimated at 33% (Table 3) and 21% (Dobie *et al*, 2006) compared to patients treated by surgery alone. When patients who did not complete chemotherapy were used as the reference group, the survival advantage was even greater at 47% (Neugut *et al*, 2006) and 55% in the current investigation (HR=0.45; 95% CI (0.33–0.60), *P*=0.005). Importantly, the same pattern of survival advantage from the completion of chemotherapy was also observed by our group in a population-based cohort of stage II colon cancer patients (unpublished). Using patients treated by surgery alone as the reference group (*n*=1362), patients who completed chemotherapy (*n*=142) showed a significant survival advantage (HR=0.63; 95% CI (0.41–0.96), *P*=0.03), whereas those who did not complete (*n*=49) showed worse survival (HR=1.27; 95% CI (0.71–2.29), *P*=0.42).

The predictors for initiation of chemotherapy were found in multivariate analysis to be younger patient age, treatment in a private hospital and preoperative colonoscopy or sigmoidoscopy (Figure 1). The first two factors are likely to reflect patients with lower comorbidities and higher socioeconomic status, respectively, whereas the third factor may represent non-emergency presentation. Interestingly, none of the reported pathological variables was predictive for the initiation of chemotherapy in stage III colon cancer. District hospitals were defined here as non-teaching and non-private institutions located in the metropolitan region of Perth. Patients treated in these hospitals showed a two-fold lower initiation rate for chemotherapy compared to teaching institutions, suggesting deficiencies in access to oncology services.

Patients with greater levels of psychosocial support and financial resources would be expected to show higher rates of chemotherapy completion. This is supported by our findings that patients with a high socioeconomic index or who were treated in teaching or private hospitals showed higher rates of completion (Table 6). Previous North American studies have shown that married status is also predictive for the completion of chemotherapy (Dobie *et al*, 2006; Neugut *et al*, 2006). It is of concern that patients treated in district and rural hospitals showed approximately half the rate of chemotherapy completion compared to patients from teaching and private hospitals (Table 6). There is clearly a need to identify the underlying reasons leading to low initiation and completion rates for the 25% of colon cancer patients treated in these centres so that equality of health provision can be improved.

There are several current and commonly used 5FU regimes ranging from protracted, continuous infusional 5FU delivery to bolus schedules. Protracted infusional regimes were developed to maximise 5FU dose intensity. They have been found to be as effective as the bolus regimens and less toxic, with less impact on quality of life (Andre *et al*, 2003; Saini *et al*, 2003; Goyle and Maraveyas, 2005). The Mayo and Roswell Park regimes are commonly used in the USA and have different safety profiles. The Mayo regime demonstrates more haematological but less gastrointestinal toxicity compared with the Roswell Park regime. In the UK and Europe the Lokich, LV5FU2 (de Gramont) or QUASAR-type regimens are commonly used and have advantages in terms of toxicity when compared with the Mayo regime, but comparable survival rates (Goyle and Maraveyas, 2005).

The other major factor likely to result in failure to complete chemotherapy is toxicity. It is well-documented that bolus schedules of 5FU cause more severe leucopenia and stomatitis in elderly patients, particularly in females (Milano *et al*, 1992; Meta-analysis Group in Cancer, 1998; Zalcberg *et al*, 1998; Popescu *et al*, 1999; Tebbutt *et al*, 2000; Sloan *et al*, 2002; Chansky *et al*, 2005). In agreement with these findings, the present study found that females were less likely to complete adjuvant chemotherapy when adjusted for other variables (Table 6), and we postulate this is due to an increased incidence of toxicity. The retrospective nature of this population-based study meant that information on treatment-related toxicity was not available. The skill and experience of the administrators of chemotherapy in the management of toxicities and the capacity to provide psychosocial support to patients may impact on the likelihood of a patient to complete their chemotherapy regime.

Many of the toxicities that lead to the termination of 5FU chemotherapy culminate around the time of the third cycle (Tebbutt *et al*, 2000). Unfortunately, steady-state plasma 5FU levels do not correlate with toxicity (Jodrell *et al*, 2001) and hence pharmacokinetic monitoring is not used to identify patients who are at increased risk of toxicity (Tebbutt *et al*, 2000). In randomised controlled trials, dose reductions are common after the first two cycles and 15–30% of patients fail to complete chemotherapy (Wolmark *et al*, 1993; O'Connell *et al*, 1997; Poplin *et al*, 2005). The relationship between systemic exposure and treatment efficacy has not been demonstrated, however, and can only be ascertained by conducting prospective randomised studies that compare a targeted dose adjustment to a fixed dose (Milano *et al*, 1994).

A novel and unexpected observation from this study was that stage III colon cancer patients who failed to complete chemotherapy showed significantly worse cancer-specific survival compared to patients treated by surgery alone (Table 3 and Figure 2). Dobie *et al* (2006) did not find a significant difference in survival between these two patient groups using 3-year cancer mortality data and Neugut *et al* (2006) did not report this comparison. It is unlikely that 5FU toxicity accounts for the increased mortality observed here as recent trials have reported a chemotherapy-related death rate of only 0.5% (Andre *et al*, 2004). The chemotherapy-related death rate in this population-based cohort of stage III colon cancer was 0.65% (3 out of 461). The 60-day mortality for patients that initiated adjuvant chemotherapy was 0.4%. This is comparable to published benchmark data for the safety of adjuvant chemotherapy in colon cancer (Katopodis *et al*, 2004).

One possible explanation is that failure to complete chemotherapy is indicative of high toxicity and this may in turn be associated with a more aggressive tumour phenotype or impairment of the host immune response. The CpG island methylator phenotype has worse prognosis (Van Rijnsoever *et al*, 2003; Ward *et al*, 2003) and is linked to the folate pathway (Kawakami *et al*, 2003). This latter pathway has been implicated in toxicity to 5FU (Pinedo and Peters, 1988).

The results of the current study have relevance for the introduction of new therapies for colon cancer. Oral fluoropyrimidines (UFT and capecitabine) mimic protracted venous infusional 5FU. Benefits of these agents include convenience, elimination of risks from indwelling central venous catheters and a different toxic profile. They are of comparable efficacy to the Mayo regime but with less toxicity (Carmichael *et al*, 2002; Douillard *et al*, 2002; Twelves *et al*, 2005). Toxicity profiles of FOLFOX and FOLFIRI (Andre *et al*, 2004; O'Connell, 2004; Allegra and Sargent, 2005) may reduce the completion of these treatments, but fewer cycles of these regimes may be as efficacious as the completed Mayo regime. A recently published safety analysis of the XELOX NO16968 study (Schmoll *et al*, 2007) found more frequent treatment discontinuations with XELOX compared to the FU/LV Mayo and Roswell Park regimes; however a comparable number of patients completed 12 weeks of therapy (88 and 82%, respectively).

In conclusion, this study confirms two recent reports (Neugut *et al*, 2006; Dobie *et al*, 2006) that stage III colon cancer patients who fail to complete 5FU adjuvant chemotherapy show worse survival than patients who completed this treatment. In addition, the current study found that patients who do not complete chemotherapy may in fact have worse survival than patients treated by surgery alone. These results have implications for the delivery of oncology services. Further research is needed to identify factors that could increase both initiation and completion rates of 5FU chemotherapy from colon cancer.

## Figures and Tables

**Table 1:** Initiation of adjuvant chemotherapy for stage III colon cancer patients according to demographic factors (*n*=1312)

**Characteristic**	**Percentage of total cases**	**Rate of chemotherapy initiation (%)**
Total	100.0	35.1
		
*Year of diagnosis*		
1994	11.2	16.3^a^
1995	11.7	32.5
1996	13.7	33.9
1997	16.1	39.6
1998	13.7	40.7
1999	13.5	47.0
2000	10.1	39.4
2001	9.9	40.0
		
*Age (years)*		
⩽55	16.8	52.5^b^
56–65	21.8	52.1
66–75	32.4	31.1
⩾76	29.0	16.8
		
*Sex*		
Male	51.6	37.0
Female	48.4	33.4
		
*Advantage/disadvantage*		
1	22.0	33.6
2	23.3	36.5
3	18.8	32.9
4	18.5	35.6
5	17.4	39.6
		
*Economic resources*		
1	22.2	32.5
2	23.1	34.0
3	17.6	32.6
4	19.1	40.2
5	18.0	39.3
		
*Hospital*		
Teaching	46.0	31.8^b^
District	11.2	21.1
Rural	14.3	33.3
Private	28.5	47.1

a *P*<0.025.

b *P*<0.0001.

**Table 2:** Initiation of adjuvant chemotherapy for stage III colon cancer patients according to pathological and clinical factors (*n*=1312)

**Characteristic**	**Percentage of total cases**	**Rate of chemotherapy initiation (%)**
Total	100.0	35.1
		
*Pathology*		
Site		
Proximal	53.5	33.0
Distal	46.5	37.5
Grade		
Well/moderate	76.1	34.8
Poor	23.9	36.1
T stage		
T1/T2	5.0	41.6
T3	71.0	35.8
T4	24.0	32.1
Nodal status		
N1	64.9	34.2
N2	35.1	36.9
Vascular invasion		
Absent	69.7	35.2
Present	30.3	35.0
Perineural invasion		
Absent	87.7	34.3
Present	12.3	41.0
Perforation		
Absent	91.8	35.1
Present	8.2	35.5
Mucinous		
Absent	73.7	35.1
Present	26.3	35.4
Lymphocytic response		
Absent	85.7	34.4
Present	14.3	39.4
		
*Clinical*		
Obstruction		
Absent	86.2	35.4
Present	13.8	33.7
Colonoscopy or sigmoidoscopy		
Yes	56.9	40.2^a^
No	43.1	28.5
Surgical case load		
Low	13.8	32.0^b^
Medium	47.4	31.2
High	38.8	41.1

a *P*<0.0001.

b *P*<0.002.

**Table 3:** Mortality hazard ratios according to number of completed cycles of adjuvant 5FU chemotherapy, multivariate-adjusted

	**Cancer-specific survival**			**Overall survival**		
**Chemotherapy (*n*)**	**HR**	**95% CI**	***P***	**HR**	**95% CI**	***P***
*None* (851)	1.00			1.00		
1–3 cycles (156)	1.37	1.09–1.72	0.008	1.09	0.88–1.35	NS
4–6 cycles (305)	0.67	0.54–0.83	<0.001	0.55	0.45–0.67	<0.001
						
1 cycle (68)	1.72	1.24–2.38	<0.001	1.26	0.93–1.71	NS
2 cycles (40)	1.19	0.79–1.80	NS	0.92	0.63–1.36	NS
3 cycles (48)	1.17	0.79–1.74	NS	1.04	0.73–1.48	NS
						
4 cycles (41)	0.74	0.46–1.20	NS	0.61	0.39–0.97	0.035
5 cycles (105)	0.77	0.56–1.07	NS	0.59	0.43–0.81	0.001
6 cycles (159)	0.53	0.39–0.72	<0.001	0.43	0.32–0.58	<0.001

CI=confidence interval; 5FU=5-fluorouracil; HR=hazard ratio.

**Table 4:** Completion of adjuvant chemotherapy for stage III colon cancer patients according to demographic factors (*n*=305)

**Characteristic**	**Percentage of total cases**	**Rate of completion (%)**
Total	100.0	66.3
		
*Year of diagnosis*		
1994	3.3	41.7
1995	9.2	68.0
1996	12.5	67.7
1997	20.0	66.3
1998	14.4	63.8
1999	15.4	69.3
2000	11.8	66.7
2001	13.4	72.0
		
*Age (years)*		
⩽55	25.2	66.4
56–65	34.4	70.5
66–75	26.9	62.1
⩾76	13.4	64.1
		
*Sex*		
Male	53.1	68.9
Female	46.9	63.3
		
*Advantage/disadvantage*		
1	16.4	52.6^a^
2	23.6	66.1
3	18.0	65.8
4	20.0	69.0
5	22.0	76.1
		
*Economic resources*		
1	13.8	45.2^b^
2	24.6	74.3
3	16.1	58.9
4	24.3	75.5
5	21.3	72.2
		
*Hospital*		
Teaching	43.6	69.3^b^
District	4.9	48.4
Rural	9.2	45.2
Private	42.3	73.3

a *P*<0.02.

b *P*<0.001.

**Table 5:** Completion of adjuvant chemotherapy for stage III colon cancer patients according to pathological and clinical factors (*n*=305)

**Characteristic**	**Percentage of total cases**	**Rate of completion (%)**
Total	100.0	66.3
		
*Pathology*		
Site		
Proximal	47.2	62.1
Distal	52.8	70.3
Grade		
Well/moderate	78.0	68.4
Poor	22.0	59.3
T stage		
T1/T2	6.6	74.1
T3	71.1	65.2
T4	22.3	67.3
Nodal status		
N1	67.2	70.4^a^
N2	32.8	58.8
Vascular invasion		
Absent	70.2	66.5
Present	29.8	65.5
Perineural Invasion		
Absent	85.6	66.1
Present	14.4	66.7
Perforation		
Absent	90.8	65.5
Present	9.2	73.7
Mucinous		
Absent	73.4	66.1
Present	26.6	66.4
Lymphocytic response		
Absent	82.0	64.6
Present	18.0	66.2
		
*Clinical*		
Obstruction		
Absent	87.5	66.8
Present	12.5	62.3
Colonoscopy or sigmoidoscopy		
Yes	69.2	70.3^b^
No	30.8	58.4
Surgical case load		
Low	11.5	60.3^c^
Medium	35.1	55.2
High	53.4	78.0

a *P*<0.02.

b *P*<0.01.

c *P*<0.001.

**Table 6:** Predictors for completion of adjuvant 5FU chemotherapy, multivariate-adjusted

	**Odds ratio**	**95% CI**	***P***
*Age of diagnosis (years)*			
⩽55	1.00		
56–65	0.98	0.55–1.76	NS
66–75	0.77	0.43–1.38	NS
⩾76	0.72	0.34–1.53	NS
			
*Sex*			
Male	1.00		
Female	0.68	0.44–1.05	0.08
			
*Site*			
Proximal	1.00		
Distal	1.37	0.84–2.24	NS
			
*Surgical caseload*			
Low	1.00		0.007
Medium	0.88	0.45–1.72	NS
High	2.06	0.99–4.25	0.05
			
*Advantage/disadvantage*			
1	1.00		
2	1.83	0.96–3.50	NS
3	1.45	0.74–2.85	NS
4	1.62	0.81–3.23	NS
5	2.18	1.07–4.44	0.032
			
*Economic resources*			
1	1.00		
2	3.55	1.79–7.04	<0.001
3	1.61	0.80–3.23	NS
4	3.20	1.58–6.48	0.001
5	2.54	1.26–5.14	0.009
			
*Hospital*			
Teaching	1.00		
Private	0.91	0.52–1.58	NS
District	0.50	0.21–1.18	NS
Rural	0.40	0.20–0.79	0.009

CI=confidence interval; NS=nonsignificant.
